# CDCA7 enhances STAT3 transcriptional activity to regulate aerobic glycolysis and promote pancreatic cancer progression and gemcitabine resistance

**DOI:** 10.1038/s41419-025-07399-1

**Published:** 2025-02-04

**Authors:** Dijie Zheng, Yazhu Deng, Lu Deng, Zhiwei He, Xinghao Sun, Yanyu Gong, Binbin Shi, Deqin Lu, Chao Yu

**Affiliations:** 1 School of Basic Medical Sciences, Guizhou Medical University, Guiyang, Guizhou Province 550025 China; 2 Department of Hepatobiliary Surgery, the Affiliated Hospital of Guizhou Medical University, Guiyang, Guizhou Province 550001 China; 3 Guizhou Provincial Institute of Hepatobiliary, Pancreatic and Splenic Diseases, Guiyang, Guizhou Province 550001 China; 4 Key Laboratory of Liver, Gallbladder, Pancreas and Spleen of Guizhou Medical University, Guiyang, Guizhou Province 550001 China; 5 Department of Vascular Surgery, the Affiliated Hospital of Guizhou Medical University, Guizhou Medical University, 550001 Guiyang, China; 6 School of Clinical Medicine, Guizhou Medical University, Guiyang, Guizhou Province 550025 China

**Keywords:** Cancer therapeutic resistance, Biomarkers

## Abstract

Cell division cycle associated 7 (CDCA7) plays a role in various malignancies, especially pancreatic cancer (PC). However, its expression pattern and functional significance in PC require further research. Therefore, this study aimed to investigate CDCA7 expression levels and biological functions in PC using in vitro and in vivo experiments. Western blotting, immunohistochemistry, and real-time polymerase chain reaction were performed to detect CDCA7 expression in PC cells and tissues. Additionally, the biological functions of CDCA7 were assessed using cell proliferation, wound healing, and Transwell assays. CDCA7 overexpression promoted PC cell proliferation, migration, and invasion, and increased resistance to the chemotherapy drug gemcitabine, possibly through enhanced aerobic glycolysis. Additionally, immunoprecipitation assay showed that CDCA7 interacted with STAT3 protein and affected the transcriptional regulation of hexokinase 2. Conclusively, targeting CDCA7 might be a promising therapeutic strategy to increase gemcitabine sensitivity by inhibiting glycolysis in PC cells.

## Introduction

Pancreatic cancer (PC) remains a serious challenge in oncology due to its high mortality rate, with only a 12% 5-year survival rate [1]. Major contributors to the poor prognosis of PC include difficulties in early detection and limited effectiveness of drug therapies [2]. Notably, due to the limited treatment options for pancreatic cancer, only approximately 20% of patients are eligible for surgical resection [3]. In addition to surgery, chemotherapy is an important treatment strategy for PC. Currently, gemcitabine is the most commonly used drug for PC chemotherapy. However, the efficacy of gemcitabine treatment in PC remains modest, with an overall response rate of less than 20%. Importantly, the low efficacy of gemcitabine is primarily due to the rapid onset of drug resistance in tumor cells, leading to inadequate therapeutic responses [4, 5]. Therefore, it is crucial to investigate the mechanisms underlying PC development and chemotherapy resistance, as well as develop efficacious targeted therapies.

Cell division cycle associated protein 7 (CDCA7) belongs to the family of cell cycle regulatory proteins. It has been identified as a c-Myc responsive gene, contributing to MYC-mediated tumorigenesis by acting as a transcriptional regulator [6, 7]. As an oncogenic gene with copy number amplification, CDCA7 exhibits high expression in various tumors and is correlated with unfavorable prognosis [8, 9]. CDCA7 promotes tumor cell proliferation, invasion, and metastasis in various cancers, including esophageal, gastric, and ovarian cancers, and regulates malignant biological processes, including inflammatory factors [10–12]. CDCA7 is a drug resistance-associated cell cycle protein that is highly expressed in paclitaxel-resistant subgroup of non-small cell lung cancer [13, 14]. However, the precise role of CDCA7 in PC progression remains unclear.

Recently, the role of metabolic reprogramming in the development of drug resistance in PC has attracted considerable attention [15]. Research findings indicate that stroma formation and the complex tumor microenvironment, including aberrant glycolytic metabolism reprogramming, are crucial factors contributing to chemotherapy resistance in PC [16]. Additionally, an increasing number of preclinical and clinical trials are being performed to develop metabolic-targeted therapies for PC [15]. Moreover, therapeutic approaches targeting metabolism holds great potential for improving unfavorable prognosis in patients with PC [15]. Overall, metabolism research not only aids in understanding cancer initiation and progression but also offers novel perspectives for PC treatment.

Signal transducer and activator of transcription 3 (STAT3) is a member of the STAT protein family. STAT3 can be phosphorylated by specific kinases, resulting in the formation of heterodimers that migrate to the nucleus where they function as transcription factors critical in various cellular processes, including cell growth and apoptosis [17–19]. For example, induction of STAT3 signaling increases tumor glycolysis and proliferation, prevents apoptosis, and promotes drug resistance [20, 21]. Additionally, FBP1 binds to STAT3, blocks the binding of STAT3 to STAT3-mediated gene promoters, inhibits glycolysis in ovarian cancer cells, and improves cisplatin resistance in ovarian cancer [22]. Based on these findings, it could be speculated that regulating glycolysis-related enzymes via STAT3 inhibition may ameliorate chemotherapy resistance [23, 24].

Therefore, this study aimed to investigate CDCA7 expression levels and functional significance in PC, focusing on its effect on STAT3. CDCA7 expression level was upregulated in PC and closely correlated with adverse clinical outcomes. Additionally, CDCA7 expression was related to the glycolysis pathway in PC cells. Moreover, high expression of CDCA7 enhanced the transcriptional activity of STAT3, which promoted hexokinase 2 (HK2) expression. Collectively, these changes increased aerobic glycolysis, ultimately promoting PC cell proliferation and invasion and gemcitabine resistance. Based on these findings, we believe that targeting CDCA7 may be a potential chemotherapy sensitization strategies for PC.

## Materials And Methods

### Bioinformatics analysis

Bioinformatics analysis was performed to verify the transcription levels of CDCA7 in the GTEx and TCGA databases using the Gene Expression Profiling Interactive Analysis (GEPIA). Additionally, we examined CDCA7 expression in cancerous and adjacent non-tumor tissues using datasets (GSE15471, GSE28735, and GSE16515) from the Gene Expression Omnibus database.

### Patient information and clinical samples

Cancer and para-cancerous specimens from 73 patients with PC were collected from the Department of Hepatobiliary Surgery, Affiliated Hospital of Guizhou Medical University. This study was approved by the Ethics Committee of the Affiliated Hospital of Guizhou Medical University, and each patient provided written informed consent.

### Cell culture

Human pancreatic cell lines HPNE, CFPAC-1, BxPC-3, MIA PaCa-2, SW1990, and PANC-1 were obtained from the American Type Culture Collection. HPNE and BxPC-3 were grown in RPMI-1640 medium supplemented with 10% FBS, whereas CFPAC-1, MIA PaCa-2, SW1990, and PANC-1 were cultivated in DMEM with 10% FBS. The cultures were maintained in a humidified atmosphere of 5% CO_2_ at 37 °C. Authentication of the cell lines was performed through STR profiling, and mycoplasma contamination was ruled out, ensuring the reliability of the experimental outcomes.

### Quantitative Real-time polymerase chain reaction (qRT-PCR)

Total RNA was extracted from clinical pancreatic tissues, adjacent non-tumor tissues, and PC cells subjected to different treatments using the RNA rapid extraction kit (YISHAN, Shanghai, China). Reverse transcription was performed to generate cDNA using PrimeScript RT (YISHEN, Shanghai, China). qRT-PCR was performed to quantify gene expression levels. The primer sequences are provided in Supplementary Table 1.

### Western blot assay

Proteins were extracted using a lysis buffer, separated using SDS-PAGE, and transferred to Millipore membranes. Thereafter, the membranes were blocked with 5% non-fat milk for a minimum of 1 h and incubated with the corresponding antibodies overnight at 4 °C. After washing with water, the membranes were incubated with affinity-labeled goat anti-mouse/rabbit antibody at 20 °C for 2 h. Protein bands were visualized using ECL reagents (Boster Biotechnology Co., Ltd.) and a chemiluminescence imaging system (Tanon). The antibody information is provided in Supplementary Table 2.

### Cell proliferation assays

Cell proliferation activity was detected using cell counting kit-8 (CCK-8), 5-ethyl-2’deoxyuridine (EdU), and plate cloning experiments. In the CCk8 experiment, cells were seeded in 96-well cells (six replicate wells per group) followed by the addition of CCK-8 reagent and further incubation for 3 h. Finally, the OD value was measured using a spectrophotometer. In the EdU experiment, cells were seeded into 24-well plates, followed by the addition of EdU reagent (Beyotime, Shanghai, China) to each well and further incubation. Finally, the ratio of edu-positive nuclei in six microscopic fields (three independent replicates) was analyzed. In the plate cloning experiment, cells were seeded in a 6-well plate and cultured. After 14 days, cells were fixed with 4% paraformaldehyde (Biosharp, Hefei, Anhui, China) for 30 min and stained with 0.25% crystal violet solution (Biosharp, Hefei, Anhui, China) for 30 min. Images of the stained cells were captured after washing with PBS.

### Wound healing assay

PC cells were seeded into a 6-well plate and cultured on growth density reached 90%. Thereafter, a wound was carefully inflicted on the cell layer using a 200 μL pipette tip, followed by the replacement of the culture medium with serum-free medium and incubation for 48 h. Images of each wound were meticulously captured at both the start (0 h) and end (48 h) of the incubation period using an inverted microscope (Olympus, Tokyo, Japan).

### Transwell assay

Briefly, PC cells were starved and seeded in the upper chamber, followed by the addition of 600 μL of 20% FBS DMEM medium to the lower chamber. After 24 h, the cells were washed with PBS, fixed with 4% paraformaldehyde for 30 min, and stained with a 0.25% crystal violet solution for another 30 min. Images were captured using an inverted microscope (Olympus, Tokyo, Japan).

### Immunohistochemistry

After fixation, embedding, sectioning, and deparaffinization, PC tissue sections were blocked with 3% H_2_O_2_ and 5% BSA. Therefore, the sections were incubated with primary antibodies targeting CDCA7, anti-Ki67, and PCNA overnight at 4 °C, followed by incubation with secondary antibodies for 2 h at room temperature. Positively stained cells and signal intensity were assessed in three randomly selected areas by two independent observers, who were blinded to the treatments.

### Animal experiment

All animal experiments were approved by the Ethics Committee of Guizhou Medical University. Forty-eight female BALB/c nude mice (6-week-old) were randomly divided into 8 groups (*n* = 6 mice/group). PC cells (2 × 10^6^) were injected into the left axilla of each mouse. Tumor volume was assessed every 7 days and calculated using the formula: Tumor volume = (length × width^2^)/2. Mice were euthanized at 42 days post-injection, and tumor tissues were harvested, weighed, and subjected to immunohistochemical analysis.

### Dual luciferase reporter assay

HK2 promoter was cloned into pGL4.10[luc2] vector. The indicated reporter vectors were transfected into PC cells together with pGL4.74[hRluc/TK] vector. After 48 hours cells were harvested. Finally, the fluorescence signals of firefly and Renilla luciferases were detected using the Dual-Luciferase Reporter Assay System (Promega, Madison, WI, USA). This process was independently repeated three times.

### Plasmid transfection and lentivirus infection

The CDCA7-overexpressing plasmid was constructed using the GV341 vector from GeneChem (Shanghai, China). Short hairpin RNA lentiviruses targeting CDCA7, STAT3, and HK2 were also acquired from GeneChem. The lentiviral particles were produced by co-transfecting the plasmids with packaging vectors and Lipo3000 (Invitrogen, MA, USA) into 293 T cells. The virus-containing supernatant was collected, filtered through a 0.45 μm filter, concentrated using PEG6000 (Sigma, #81253), and then resuspended in PBS before being aliquoted for future transfections. Infected cells were selected with puromycin for 72 h. The shRNA sequences are listed in Supplementary Table 3.

### Immunofluorescence assay

After exposure to various treatments, PC cells were seeded into a 24-well plate, fixed, permeabilized, blocked, and incubated with primary antibodies against CDCA7 and STAT3 at 4 °C for 10 h. Thereafter, the cells were incubated with secondary antibodies in the dark at room temperature for 2 h. After washing with PBS, cell nuclei were counterstained with 4’,6-diamidino-2-phenylindole, and visualization and quantification of target protein expression were performed using fluorescence microscopy.

### Immunoprecipitation, silver staining, and mass spectrometry analysis

Proteins were extracted from PC cells and incubated overnight at 4 °C with anti-CDCA7 antibody. Thereafter, protein A + G beads (Beyotime, Shanghai, China) were added and incubated for 2 h under constant rotation. The beads were collected using a magnetic rack and boiled after adding 1× loading buffer. Protein analysis was conducted using western blotting, silver staining, or mass spectrometry (MS). Silver staining was performed according to the manufacturer’s instructions.

### Glucose uptake, ATP production rate, and lactic acid production assays

PC cells at the log-phase were plated in 96-well plates. Glucose uptake, ATP levels, and lactate production were measured using the Glucose Assay Kit (Absin, abs580025), ATP Assay Kit (Sigma, MAK190), and Lactate Assay Kit (Sigma, MAK064), respectively, according to the manufacturers’ protocols.

### Construction of Orthotopic Tumor Models

The orthotopic pancreatic cancer tumor studies were conducted using C57BL/6 J mice. Female mice were randomly divided into 8 groups, with 5 mice per group. At the age of 8 weeks, the mice underwent orthotopic pancreatic cancer tumor induction. A suspension of 1 × 10^6^ PANC-02-Luc cells was harvested and injected into the pancreatic tail via a minimal laparotomy. Twenty-one days following the injection, high-resolution ultrasound imaging was conducted on each mouse to verify tumor establishment.

### Extracellular acidification rate

To assess PC cell metabolism, cells were seeded in Seahorse XF96 plates (Seahorse, Cat No. 101085-004) at a density of 3.5 × 10^4^ cells/well until cell confluence reached 80%. After rinsing with PBS, glycolysis and extracellular acidification rate (ECAR) were measured using Seahorse Glycolytic Stress Test Kit (Seahorse, Cat No. 103020-100) and Seahorse XFe96 Analyzer, respectively. The analysis was performed on three independent replicates.

### Statistical analysis

All statistical analyses were performed using SPSS (version 23.0; IBM Corp., Armonk, NY, USA). Data are expressed as mean ± standard deviation. Significant differences were determined using Student’s *t*-test for two group comparison or one-way analysis of variance for multi-group comparison. Overall survival was determined using the Kaplan-Meier method. Statistical significance was set at *p* < 0.05.

## Result

### CDCA7 is highly expressed in PC tissues and cells

**Fig. 1:**
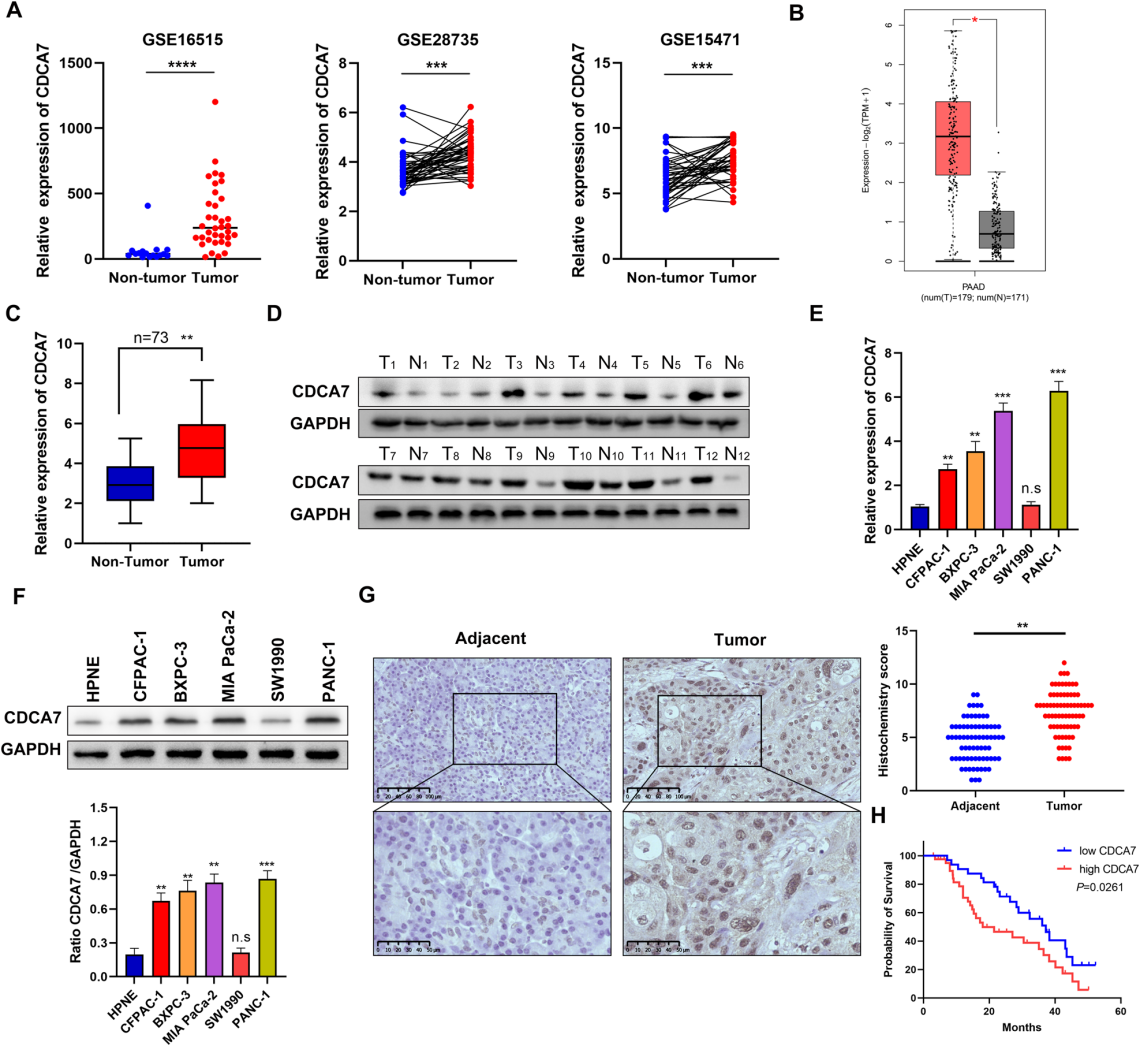
CDCA7 is highly expressed in PC tissues and cells. **A** Analysis of datasets (GSE16515, GSE15471, GSE28735) from the GEO database showed that CDCA7 has a higher expression in PC tissues than in normal or non-tumor tissues. **B** CDCA7 mRNA expression levels were analyzed in 179 PC tissue samples and 171 normal pancreatic tissue samples using data from the GTEx and TCGA databases. **C**, **D** qRT-PCR and western blotting were performed to detected CDCA7 mRNA and protein levels, respectively, in PC tissues and para-cancerous tissues. **E**, **F** qRT-PCR and western blotting detected CDCA7 mRNA and protein expression levels, respectively, in HPNE and PC cells. **G** IHC confirmed CDCA7 expression levels in PC tissue and adjacent tissue. **H** Kaplan-Meier survival curves were generated to elucidate the survival outcomes in patients with PC with varying CDCA7 expression levels. *P < 0.05, **P < 0.01, ***P < 0.001.

Expression profile analysis of datasets (GSE16515, GSE28735, and GSE15471) downloaded from the Gene Expression Omnibus database showed that CDCA7 mRNA expression was higher in PC tissues than in adjacent para-cancerous tissues (Fig. 1A). Similarly, CDCA7 expression was higher in 179 PC samples than in 171 normal pancreatic tissues in the Cancer Genome Atlas (TCGA) database (Fig. 1B). Additionally, we examined CDCA7 mRNA and protein expression in clinical tissue samples using qRT-PCR and western blotting, respectively. Notably, CDCA7 expression was markedly higher in PC tissues than in adjacent tissues at both the mRNA and protein levels (Fig. 1C, D). Moreover, we examined CDCA7 mRNA and protein levels in human pancreatic normal ductal epithelial (HPNE) cells and five human PC cell lines (CFPAC-1, BxPC-3, MIA PaCa-2, SW1990, and PANC-1). CDCA7 mRNA and protein levels were higher in all PC cell lines (except in SW1990) than in HPNE cells (Fig. 1E, F). Similarly, immunohistochemistry (IHC) showed that CDCA7 expression was higher in PC tissues than in adjacent tissues (Fig. 1G). Moreover, Kaplan-Meier survival analysis showed that patients with elevated levels of CDCA7 had markedly lower survival rate than those with low CDCA7 expression (Fig. 1H).

### CDCA7 promotes the proliferation, migration, and invasion of PC cells

**Fig. 2:**
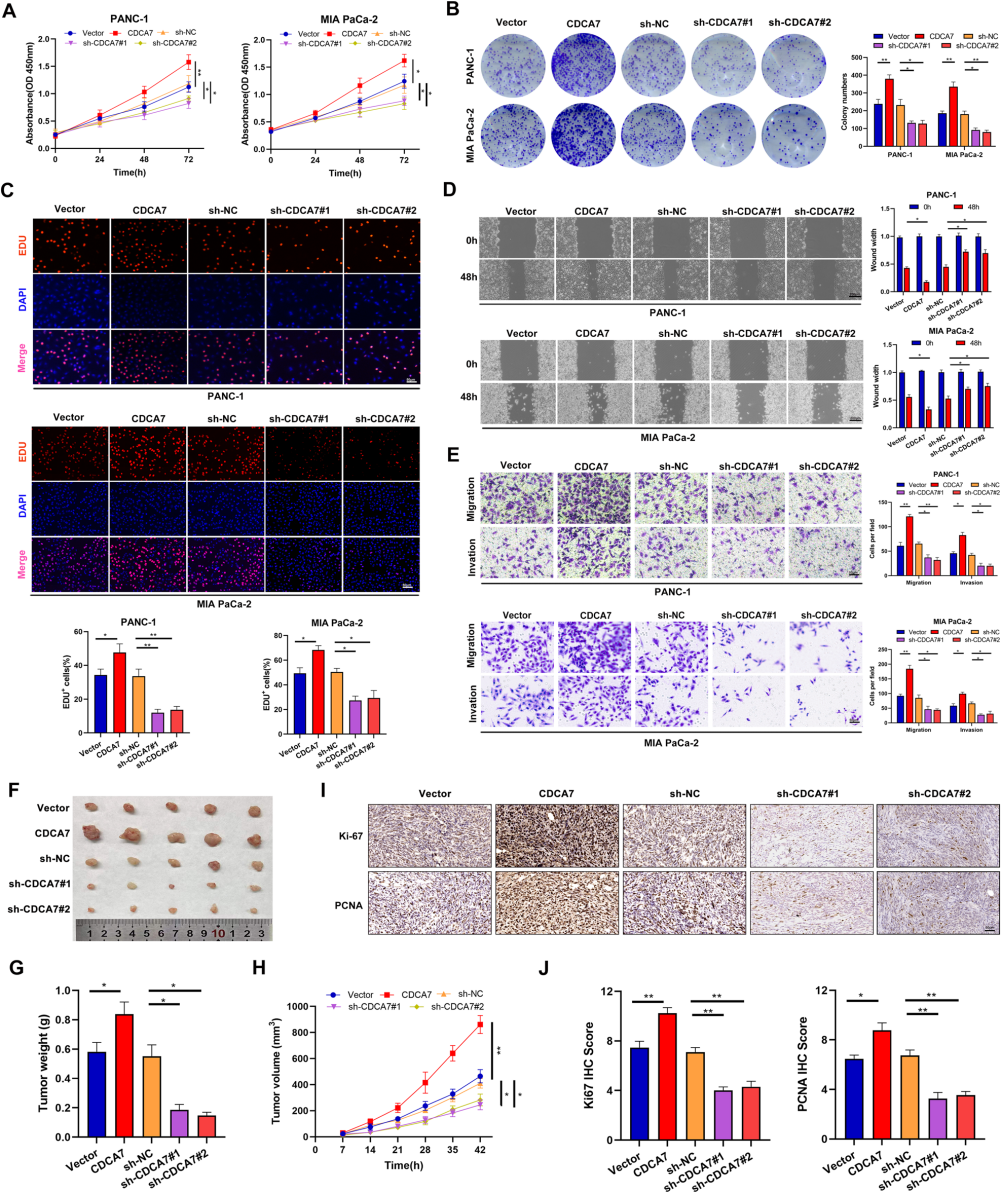
CDCA7 promotes the proliferation, migration, and invasion of PC cells. **A**–**C** Effects of CDCA7 overexpression and knockdown on the proliferation of PC cells, detected using CCK-8 assay (**A**), plate clone (**B**), EDU (**C**) (scale bar: 50 μm). **D**, **E** Wound healing and Transwell assays were used to detect changes in the migratory and invasive abilities of PC cells following CDCA overexpression or knockdown. **F**–**H** Establishment of xenograft tumor model, **F** Resection of subcutaneous tumors 42 days after transplantation of PC cells **G** Tumor weight. **H** Tumor volume. **I**, **J** IHC was performed to detect Ki-67 and PCNA expression in tumor tissues. *P < 0.05, **P < 0.01, ***P < 0.001.

To elucidate the functional roles of CDCA7, the PC cell lines PANC-1 and MIA PaCa-2 were transfected with various plasmids to generate CDCA7 overexpression and knockdown models. CDCA7 overexpression or knockdown efficiency was confirmed using western blotting and qRT-PCR (Supplementary Fig. 1A-B). CCK-8, colony formation, and EdU assays showed that CDCA7 overexpression significantly promoted the proliferation of PC cells, whereas CDCA7 knockdown had the effect t (Fig. 2A–C). Similarly, CDCA7 overexpression significantly upregulated the proliferation of SW1990 cells (Supplementary Fig. 1C−E). Moreover, wound healing (Fig. 2D) and Transwell (Fig. 2E) assays indicated that CDCA7 overexpression promoted the migratory and invasive abilities of PC cells, whereas CDCA7 knockdown had the opposite effects. Similarly, CDCA7 overexpression increased the migratory and invasive abilities of SW1990 cells (Supplementary Fig. 1F, G). To confirm whether the cellular phenotypic changes were caused by CDCA7, we established distinct groups, including sh-NC, sh-CDCA7, and sh-CDCA7 + CDCA7_Res groups. Significantly, sh-mediated knockout of CDCA7 genes suppressed PC cell proliferation, migration, and invasion. However, reintroducing the CDCA7 gene successfully restored cellular functions (Supplementary Fig. 1H-L). To evaluate the role of CDCA7 on PC cells in vivo, mice were injected with PANC-1 cells to construct a subcutaneous tumor model (Fig. 2F). CDCA7 overexpression promoted tumor growth, whereas CDCA7 knockdown inhibited tumor growth, as evidenced by tumor volume and weight (Fig. 2G, H). Additionally, IHC showed that CDCA7 level was positively correlated with the expression of the proliferation indicators Ki-67 and PCNA in subcutaneous tumor tissues derived from mice (Fig. 2I, J). Furthermore, we developed an orthotopic pancreatic tumor model in C57BL/6 mice. Bioluminescence imaging (BLI) showed a significant increase in signal intensity in the *cdca7* overexpression group compared with that in the control group (Supplementary Fig. 1M).

### CDCA7 enhances glycolysis in PC cells and promotes gemcitabine resistance

**Fig. 3:**
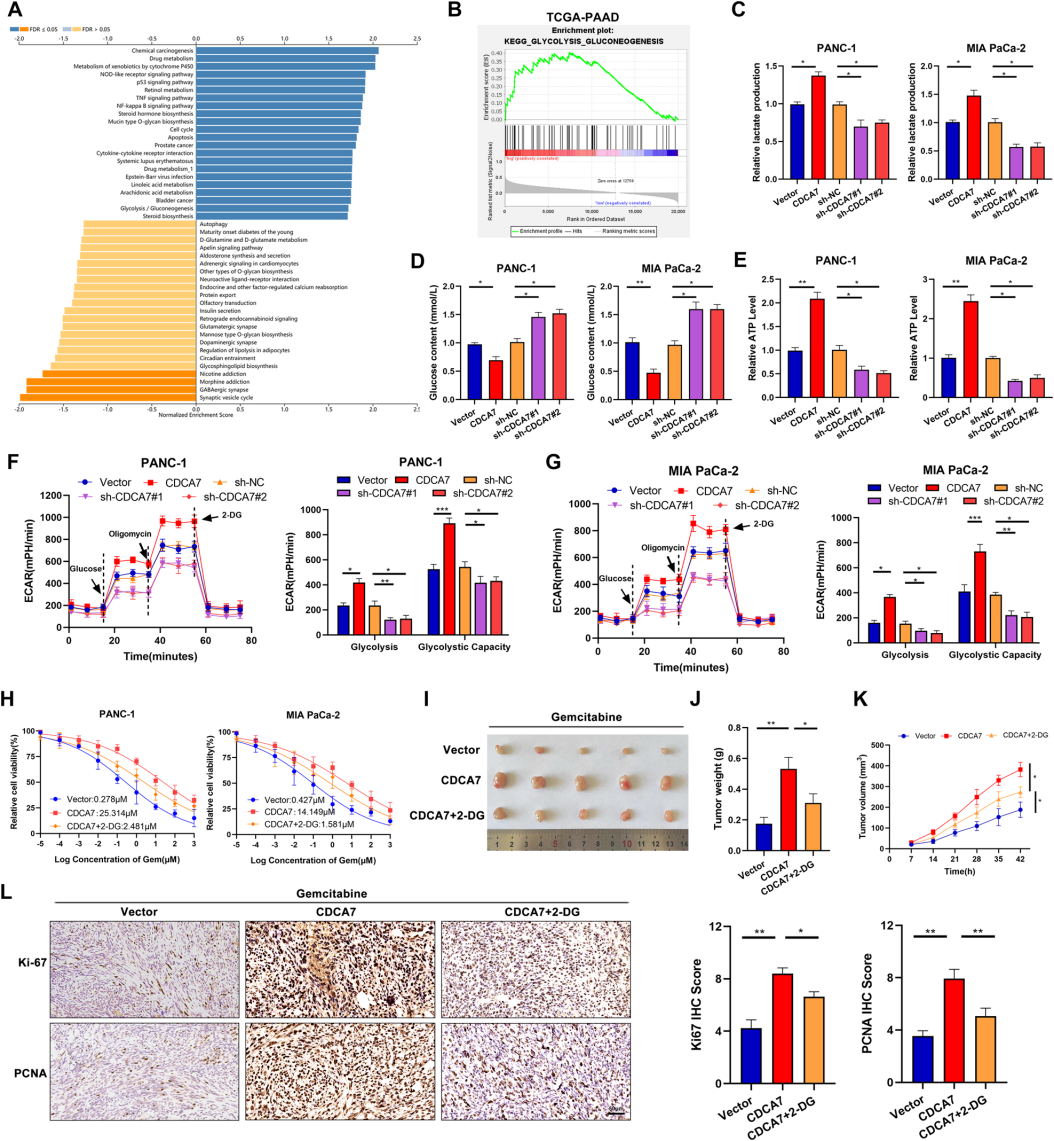
CDCA7 enhances glycolysis in PC cells and promotes gemcitabine resistance. **A**, **B** Samples were stratified into high (≥ 50%) and low (< 50%) expression groups based on CDCA7 expression levels, and single-gene Gene Set Enrichment Analysis (GSEA) was conducted to identify pathways associated with CDCA7 upregulation and downregulation. **C**–**E** Measurement of lactate (**C**), glucose (**D**) and ATP (**E**) levels in PC cells following CDCA7 overexpression or knockdown. **F**, **G** Extracellular acidification rate (ECAR) indicates aerobic glycolysis level in PC cells following CDCA7 overexpression or knockdown. **H** CCK8 assay was performed detects the sensitivity of PC cells in the control, CDCA7 overexpression, and CDCA7 + 2-DG groups to gemcitabine. **I** Establishment of xenograft tumor model and isolation of the subcutaneous tumor 42 days later. **J**, **K** Tumor weight and volume. **L** IHC was performed to detect Ki-67 and PCNA in tumor tissues in each group. *P < 0.05, **P < 0.01, ***P < 0.001.

Gene set enrichment analysis (GSEA) of dataset from the TCGA database indicated that the glycolysis pathway was positively enriched in PC tissues (Fig. 3A, B). To investigate the role of CDCA7 in metabolism in PC cells, extracellular glucose levels, extracellular lactate levels, and intracellular ATP levels were measured. Notably, the CDCA7 overexpression group showed decreased glucose content in the culture medium, increased lactic acid production, and elevated intracellular ATP levels. In contrast, the CDCA7 knockdown group exhibited increased glucose content, decreased lactic acid production, and downregulated ATP levels (Fig. 3C–E). Additionally, we examined ECAR in the two PC cell lines using Seahorse energy metabolism analyzer. Although CDCA7 upregulation promoted glycolysis, its downregulation suppressed glycolysis in both cells (Fig. 3F, G). Research evidence suggests that drug-resistant tumor cells possess higher glycolytic activity than sensitive cells. Therefore, modulating the glycolytic pathway could be an effective strategy to overcome tumor resistance to chemotherapy [25]. To confirm this hypothesis, PC cells overexpressing CDCA7 were treated with 2-DG, a critical inhibitor of glycolysis. Importantly, 2-DG treatment significantly enhanced the sensitivity of the cells to gemcitabine (Fig. 3H). Consistent with the in vitro results, in vivo experiments showed CDCA7 overexpression promoted tumor resistance to gemcitabine, a phenomenon that is reversed following 2-DG treatment (Fig. 3I–K). IHC showed a marked decrease in the expression of Ki-67 and PCNA in CDCA7-overexpressing cells treated with 2-DG (Fig. 3L). Moreover, the BLI of orthotopic pancreatic tumor showed decreased signal intensity in the *cdca7* + 2-DG group compared with that in *cdca7*overexpression group (Supplementary Fig. 1N).

### CDCA7 interacts with STAT3

**Fig. 4:**
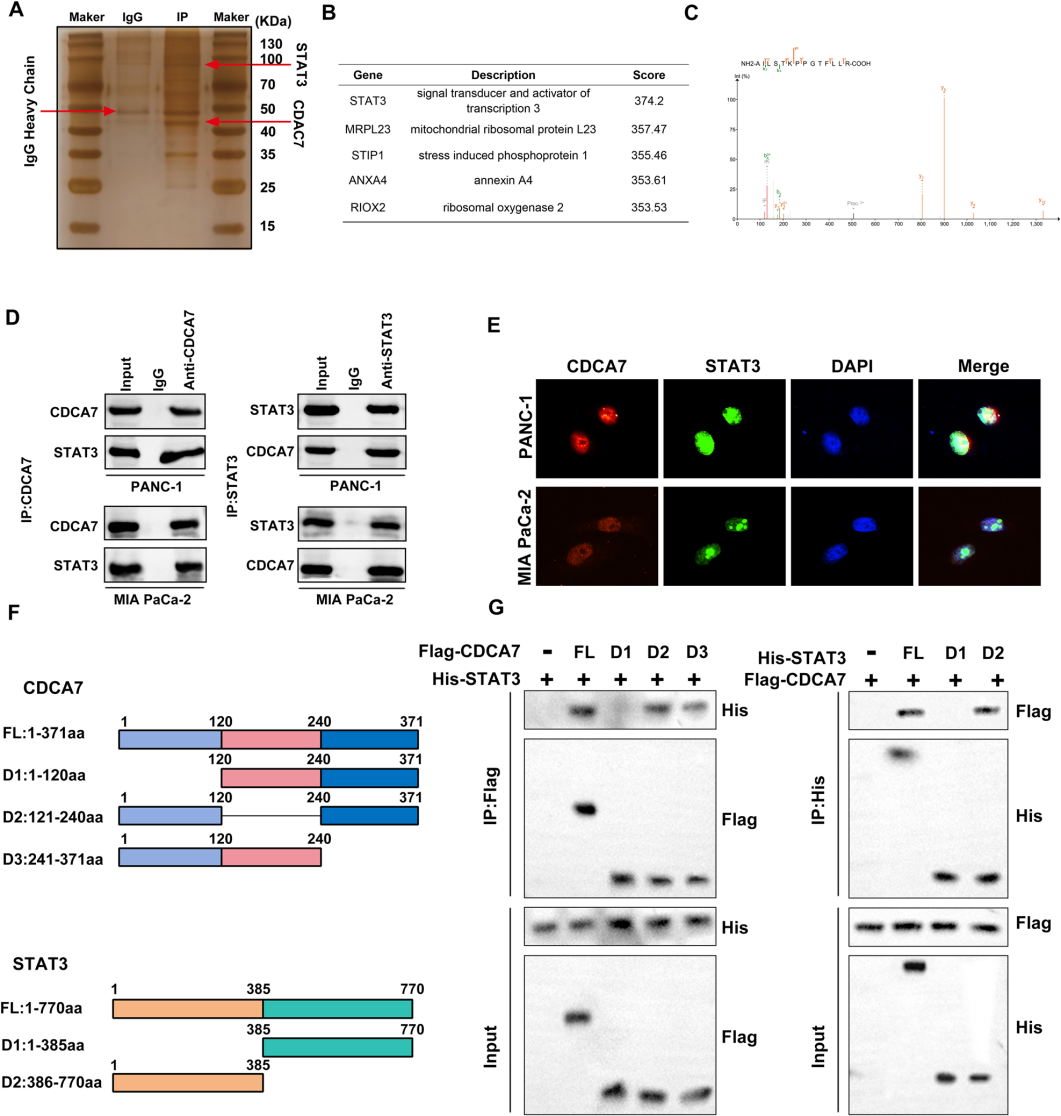
Interaction between CDCA7 and STAT3. **A** Detection of different protein bands using silver staining method following CDCA7 overexpression in PANC-1 cells. **B** Mass spectrometry analysis of five proteins coprecipitated with CDCA7. **C** A distinct CDCA7 polypeptide was identified from the protein lysate of anti-CDCA7 immunoprecipitated PANC-1 cells using two-dimensional LC-MS/MS mass spectrometry. **D** Co-immunoprecipitation assay to detect the interaction between CDCA7 and STAT3. **E** CDCA7 co-localizes with STAT3 in PC cells. **F** Design of CDCA7 and STAT3 mutants. **G** Immunoprecipitation and western blotting were performed to detect the interaction between flag-tagged truncated CDCA7 protein and his-tagged STAT3 protein in HEK 293 T. *P < 0.05, **P < 0.01, ***P < 0.001.

Considering that CDCA7 has been shown to inhibit glycolysis in PC cells, we examined the specific regulatory mechanism of CDCA7 in glycolysis. A silver staining assay was conducted to discern differential protein bands within the immunoprecipitated complexes, followed by mass spectrometry (Fig. 4A, B). Figure 4C shows the secondary protein spectrum of CDCA7. Co-immunoprecipitation assay demonstrated a possible interaction between CDCA7 and STAT3 (Fig. 4D). Immunofluorescence co-localization assay showed that CDCA7 and STAT3 were colocalized in the nucleus (Fig. 4E). To identify the binding sites of CDCA7 and STAT3, we generated a several shortened CDCA7 and STAT3 mutants (Fig. 4F). Notably, the D1 (1-120aa) domain of CDCA7 and the D1 (1-385aa) domain of STAT3 are necessary and sufficient for direct interaction (Fig. 4G).

### STAT3 promotes pancreatic cancer progression

To assess the impact of STAT3 on the progression of PC cells, we generated STAT3 overexpression and knockdown PC cell lines. Western blotting and qRT-PCR confirmed successful STAT3 knockdown and overexpression (Supplementary Fig. 2A, B). Cellular functional assays revealed that STAT3 knockdown significantly inhibited PC cell proliferation (Supplementary Fig. 2C–E). To determine if the observed decrease in cell proliferation was attributable to STAT3, we performed reconstitution experiment. Importantly, reintroducing the full-length STAT3 construct into STAT3 knockdown cells effectively restored the proliferation rate of PC cells (Supplementary Fig. 2F–H). Wound healing and Transwell assays demonstrated that STAT3 inhibited the migratory and invasive abilities of PC cells (Supplementary Fig. 2I, J). To examine the effects of STAT3 on metabolism in PC cells, we measured glucose and lactate level in the extracellular environment and ATP levels within the cells. Compared with those in the control group, STAT3 knockdown elevated glucose levels, reduced lactate production, and downregulated ATP levels (Supplementary Fig. 2K–M). Additionally, ECAR was notably lower in the STAT3 knockdown group than in the control group (Supplementary Fig. 2N). Moreover, STAT3 knockdown enhanced the sensitivity of PC cells to gemcitabine (Supplementary Fig. 2O).

### CDCA7 promotes PC cell progression and gemcitabine resistance by targeting STAT3 to regulate glycolysis levels

**Fig. 5:**
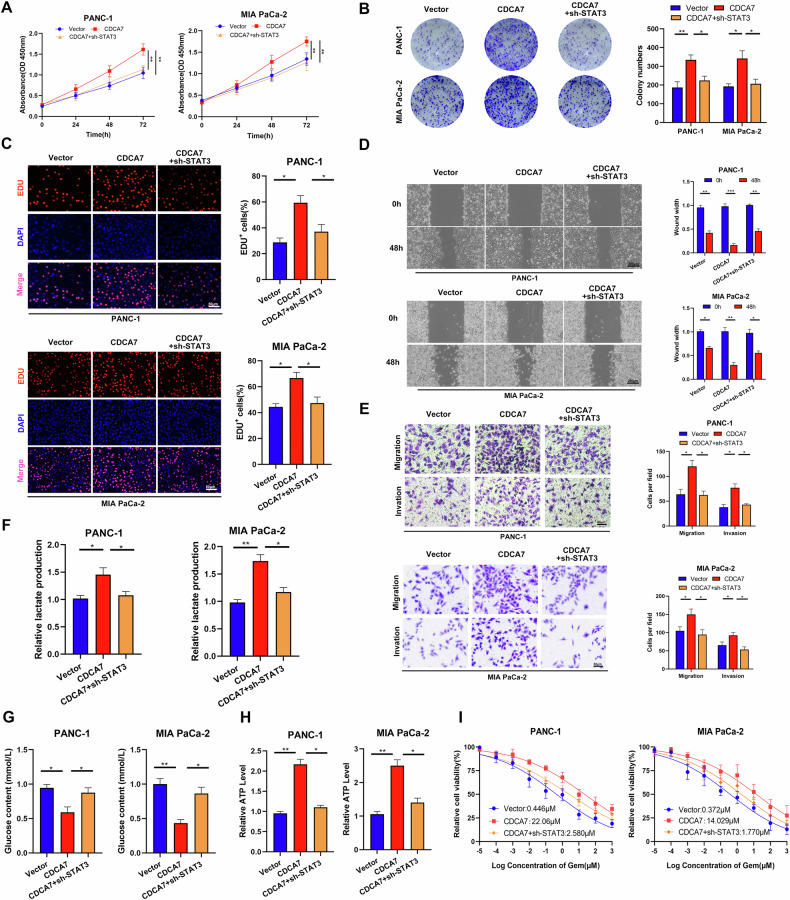
CDCA7 promotes PC cell progression and gemcitabine resistance by targeting STAT3 to regulate glycolysis levels. PANC-1 and MIA PaCa-2 were transfected with or without plasmids to generate Vector, CDCA7 overexpression, and CDCA7 + sh-STAT3 groups. **A** CCK-8, (**B**) plate clone, (**C**) EDU assays (scale bar: 50 μm). **D** Wound healing (scale bar: 200 μm) and (**E**) Transwell assays. **F**–**H** Detection of extracellular glucose and lactate levels and intracellular ATP levels in PC cells. **I** CCK-8 assay was performed to detect the sensitivity of each group to gemcitabine. *P < 0.05, **P < 0.01, ***P < 0.001.

In the present study, we examined the effect of STAT3 on the proliferation and invasive ability of PC cells overexpressing CDCA7. CCK8, colony formation, and EDU assays showed that STAT3 knockdown inhibited CDCA7-mediated PC cell proliferation (Fig. 5A–C). Additionally, wound healing and Transwell assays revealed that STAT3 knockdown attenuated CDCA7 overexpression-induced upregulation of PC cell migration and invasion (Fig. 5D, E). Moreover, STAT3 knockdown increased glucose levels and decreased both lactate production and intracellular ATP levels in PC cells overexpressing CDCA7 compared with those in the CDCA7 overexpression group (Fig. 5F–H). Similarly, STAT3 knockdown attenuated CDCA7 overexpression-induced gemcitabine resistance in PC cells to a certain degree (Fig. 5I). Collectively, these results indicate that CDCA7 promotes PC progression and gemcitabine resistance possibly through STAT3-mediated regulation of aerobic glycolysis.

### CDCA7-STAT3 axis enhances HK2 expression and promotes glycolysis

**Fig. 6:**
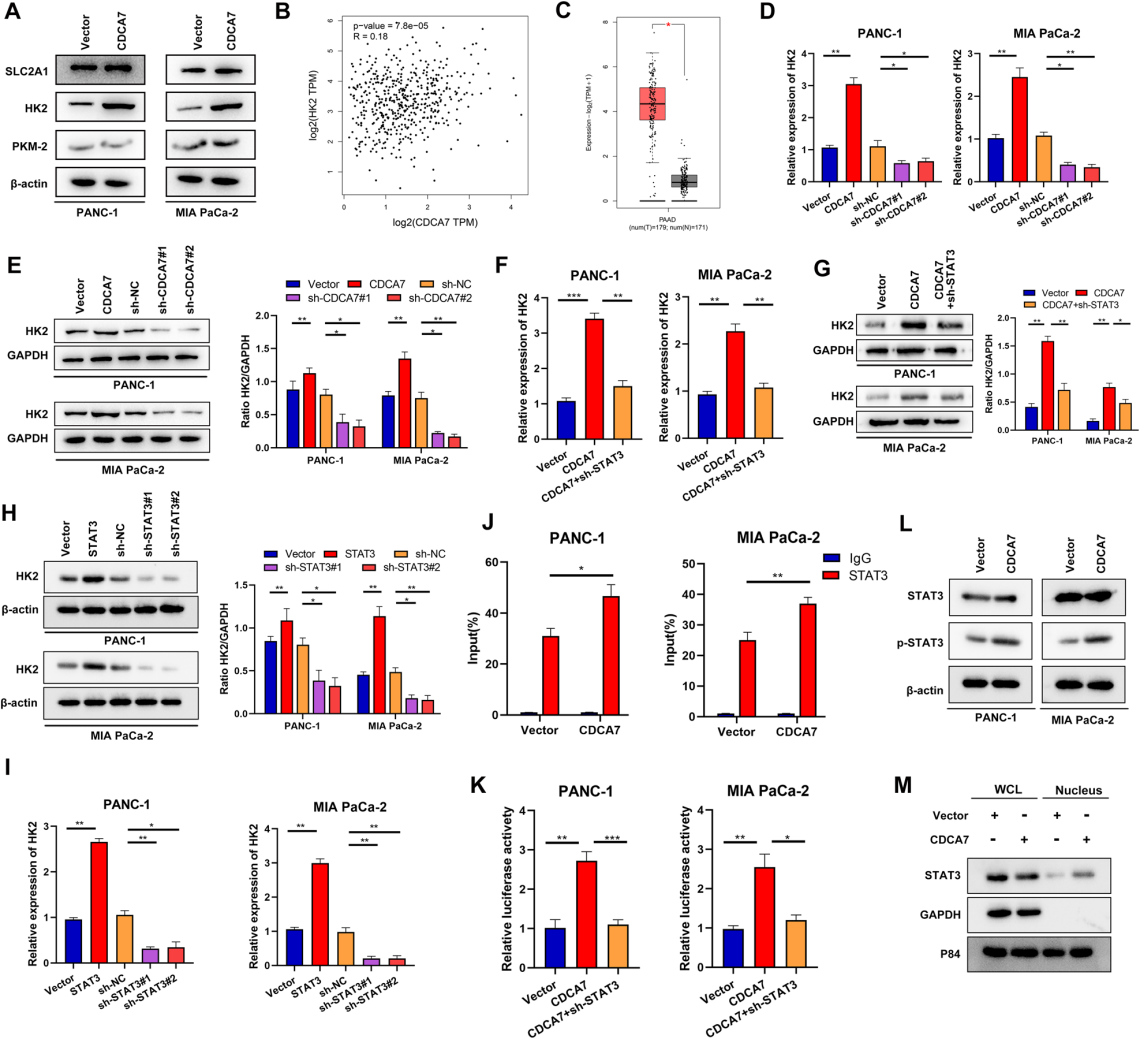
CDCA7-STAT3 axis enhances HK2 expression and promotes glycolysis. **A** Western blotting detected changes in key enzymes during glycolysis. **B** Correlation between CDCA7 and HK2 in the GEPIA database. **C** HK2 mRNA expression in PC tissue samples and normal pancreatic tissue samples using data from the GTEx and TCGA databases. **D**, **E** qRT-PCR and western blotting were performed to detect HK2 expression after CDCA7 knockdown or overexpression. **F**, **G** HK2 expression was detected in CDCA7 overexpressing cells transfected with or without sh-HK2 using qRT-PCR and western blotting. **H**, **I** Western blotting and qRT-PCR were performed detect HK2 expression following STAT3 knockdown or overexpression. **J** CHIP-qPCR detected STAT3 and HK2 gene. **K** Luciferase activity was detected PC cells co-transfected with CDCA7 and sh-STAT3. **L** STAT3 phosphorylation level was detected using western blotting. **M** Western blotting was performed to detect Whole cell lysates (WCLs) and nuclear expression of STAT3 protein. *P < 0.05, **P < 0.01, ***P < 0.001.

To elucidate alterations in glycolysis levels, we conducted CDCA7 overexpression experiments and assessed changes in key glycolytic enzymes. Western blotting showed that HK2 protein levels were significantly upregulated in the CDCA7 overexpression group (Fig. 6A). Additionally, the GEPIA database showed a positive correlation between CDCA7 and HK2 (Fig. 6B). Moreover, HK2 expression was upregulated in PC samples in the TCGA database (Fig. 6C). qRT-PCR and western blotting showed that CDCA7 overexpression or knockout influenced the expression levels of HK2 (Fig. 6D, E). Considering that CDCA7 interacts with STAT3 to regulate glycolysis, we examined changes in HK2 expression in PC cells overexpressing CDCA7 after STAT3 knockdown. qRT-PCR and western blotting indicated that STAT3 suppression abrogated the regulatory impact of CDCA7 on HK2 expression (Fig. 6F, G). Similarly, the regulation of HK expression by STAT3 was confirmed using qRT-PCR and western blotting (Fig. 6H, I). CHIP-qPCR showed that the binding of STAT3 to HK2 promoter was significantly enhanced in CDCA7-overexpressing PC cells (Fig. 6J). Moreover, luciferase assay showed that high expression of CDCA7 upregulated STAT3 transcriptional activity and promoted HK2 transcription (Fig. 6K). To elucidate the underlying mechanism by which CDCA7 enhances the DNA binding capacity of STAT3, we investigated changes in STAT3 phosphorylation following CDCA7 overexpression. Notably, CDCA7 enhanced STAT3 phosphorylation in PC cells (Fig. 6L). Additionally, western blotting revealed a significant increase in the levels of STAT3 protein within the nucleus following CDCA7 overexpression in PANC-1 cell (Fig. 6M). In summary, CDCA7 augments STAT3 activity within the HK2 promoter region, thereby stimulating HK2 transcription and intensifying glycolytic activity in PC cell.

### STAT3 regulates HK2 to promote the progression and drug resistance of PC

**Fig. 7:**
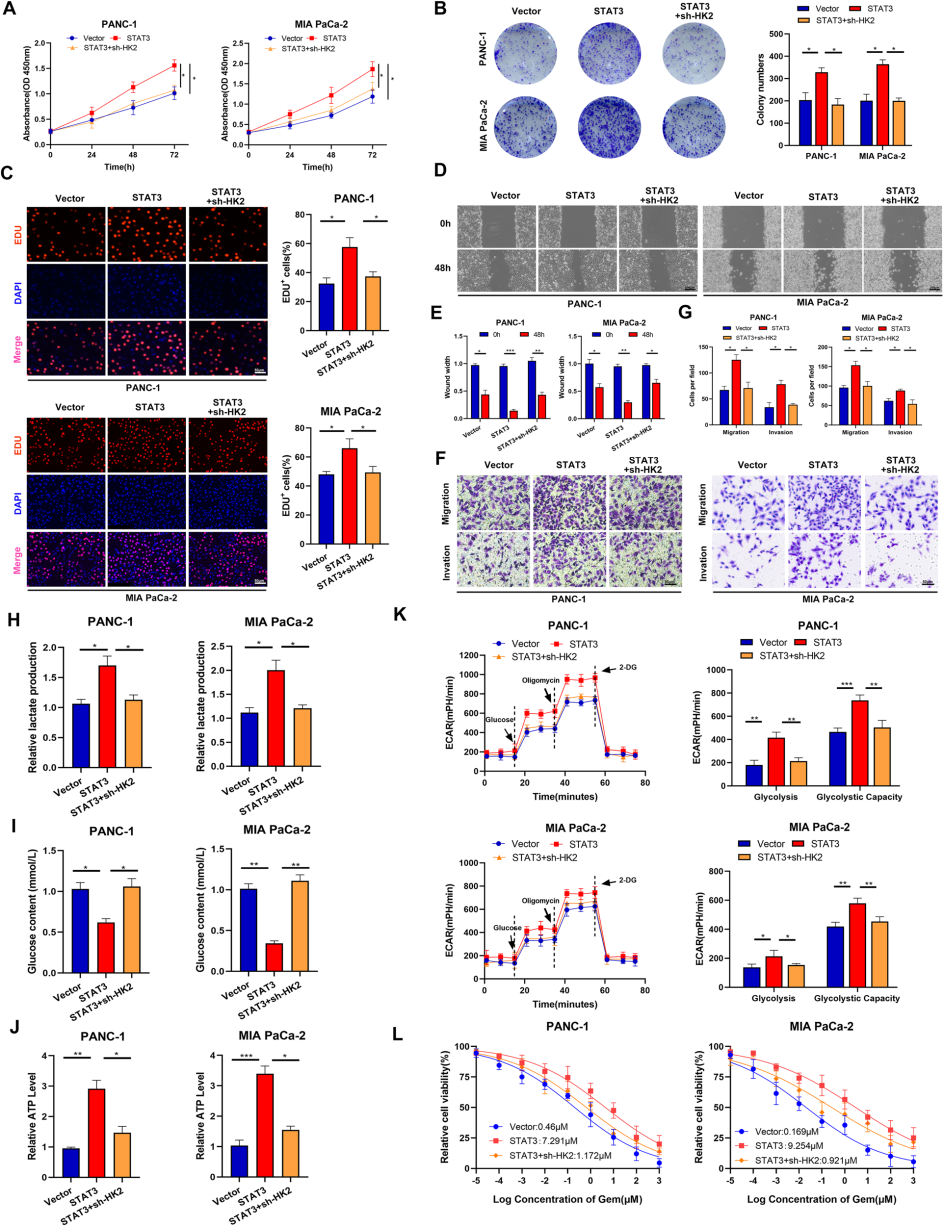
HK2 downregulation reverses STAT3-mediated PC progression. **A**–**C** CCK-8, plate cloning, and EDU assays were performed to examine cell proliferation in each group. **D**–**G** Migratory and invasive abilities of cells in each group were determined using scratch and Transwell assays. **H**–**J** Lactate (**H**), glucose (**I**), and ATP levels (**J**) in each group. **K** Aerobic glycolysis was assessed in each group, based on ECAR. **L** CCK-8 assay was performed to examine the resistance of PC cells to gemcitabine in each group. *P < 0.05, **P < 0.01, ***P < 0.001.

Western blot and qRT-PCR confirmed successful HK2 knockdown in PC cells (Supplementary Fig. 3A, B). To determine whether STAT3 facilitates PC progression through HK2, we knocked down HK2 in STAT3-overexpressing PC cells. Compared with those in the STAT3 overexpression group, co-transfection with OE-STAT3 and sh-HK2 significantly reduced the proliferation and migration and invasive abilities of PC cells (Fig. 7A–G). Similar trends were observed in glycolysis level and ECAR (Fig. 7H–K). HK2 knockdown partially ameliorated STAT3 overexpression induced gemcitabine resistance (Fig. 7L). Collectively these suggest that STAT3 may promote aerobic glycolysis to enhance PC cell progression and gemcitabine resistance via HK2.

## Discussion

PC is currently one of the deadliest types of cancer, with poor treatment outcomes. Notably, surgical opportunities are missed because of the insidious onset and late detection of PC. Moreover, the highly dense nature and metabolic complexity of the tumor tissue result in poor drug treatment. Although combined radiotherapy and immunotherapy has shown poor outcomes, research evidence suggests that metabolic reprogramming may play a key role in PC carcinogenesis, progression, treatment, and prognosis. Moreover, metabolic reprogramming is intricately linked to chemotherapy, radiotherapy, and immunotherapy, ultimately contributing to unfavorable prognostic outcomes [15]. Glycolysis reprogramming caused by dense tissue of PC is one of the metabolic characteristics [26]. Therefore, exploring and analyzing the mechanism of aerobic glycolysis in PC may have important clinical value.

CDCA7 is a member of the CDCA family, and its abnormal upregulation in tumors regulate the malignant biological behavior. CDCA7 promotes TGF-β-induced epithelial-mesenchymal transition to promote tumor progression by transcriptionally regulating Smad4/Smad7 in esophageal cancer cells [10]. Chen et al. showed that CDCA7 promotes zeste homolog 2 (EZH2) expression to promote ovarian cancer angiogenesis [12]. Yu et al. found that CDCA7 regulates inflammatory responses through the TLR4/NF-κB signaling pathway in gastric adenocarcinoma [11]. In the present study, analysis of public databases, IHC, and qRT-PCR showed that CDCA7 expression was significantly higher in human PC tissues than in adjacent non-cancerous tissues, and is significantly negatively correlated with prognosis and survival. In vivo and in vitro experiments confirmed that CDCA7 overexpression promoted the proliferation of PC cells. Moreover, the reprogramming of glucose, amino acid, and lipid metabolism, along with metabolic interplay within the tumor microenvironment, plays a pivotal role in driving the progression of pancreatic tumors. Research evidence indicates a correlation between gemcitabine resistance and metabolic pathways involving glucose, amino acids, and lipids [27]. Zhao et al. showed that gemcitabine-resistant PC cell lines exhibited high aerobic glycolysis and low ROS levels, which may contribute to chemotherapy resistance [28]. Additionally, Surendra et al. showed that elevated expression of the transmembrane protein MUC1 activated and stabilized HIF-1α, thereby promoting glycolysis and chemotherapy resistance [29]. In this study, bioinformatics analysis showed that CDCA7 was enriched in the glycolysis pathway. In vivo and in vitro experiments showed that CDCA7 overexpression increased glycolysis and promoted gemcitabine resistance in PC cells. However, treatment with a key inhibitor of glycolysis (2-DG) reversed the effects of CDCA7 overexpression.

Importantly, CDCA7 is a protein related to transcription factors [10]. To verify the molecular mechanism by which CDCA7 regulates glycolysis and affects drug resistance, we performed mass spectrometry and immunoprecipitation assay. Immunoprecipitation and colocalization assays showed that CDCA7 interacted with STAT3, with both proteins mainly colocalized in the nucleus. Further analysis using truncated bodies confirmed the interaction between the D1 (1-120aa) domain of CDCA7 and the D1 (1-385aa) domain of STAT3. STAT3 is a signal transducer and transcriptional activator that is often phosphorylated and activated to function as a transcriptional activator. For example, enhanced STAT3 transcriptional signal can increase glycolysis and promote tumor progression [20]. STAT3 directly regulates the transcription of solute carrier family 2 member 1 (SLC2A1) to promote glycolysis [30]. Li et al. showed that STAT3 overexpression regulated the expression of key enzymes in glycolysis, leading to a direct increase in glucose consumption and lactate formation [31]. Consistent with the above findings, western blotting and qRT-PCR showed that CDCA7-STAT3 interaction affected HK2 expression in PC cells. HK2, a key enzyme that regulates the first step of glycolysis, is overexpressed in several tumors and upregulates glycolysis to promote gemcitabine drug resistance [32–34]. Dual-luciferase assay showed that CDCA7 overexpression increased the transcriptional activity of STAT3. CHIP-qPCR showed a significant increase in the binding of STAT3 to HK2 promoter in CDCA7-overexpressing PC cells, upregulating the transcription and protein levels of HK2 and promoting the resistance of PC cell lines to gemcitabine. Notably, HK2 downregulation partly ameliorated STAT3 overexpression-induced increase in aerobic glycolysis and PC cell proliferation, migration, and invasion, and suppressed gemcitabine resistance. Collectively, these results suggest that the CDCA7/STAT3/HK axis may play a key role in aerobic glycolysis and PC progression and influence the sensitivity of PC to gemcitabine.

Conclusively, CDCA7 acts as an oncogene in PC and regulates aerobic glycolysis by enhancing the transcriptional activity of the transcription factor STAT3 and promoting the transcription of the key glycolysis enzyme HK2, which may contribute to drug resistance in PC cells. Therefore, targeting CDCA7 could be a potential therapeutic strategy for increasing the sensitivity of PC cells to gemcitabine.

## Supplementary information

supplementary information

Original Data1

Original Data2

## Data Availability

The datasets produced and examined during the present investigation can be obtained from the corresponding author upon a reasonable inquiry.
