# Supplementary material for: CDCA7 enhances STAT3 transcriptional activity to regulate aerobic glycolysis and promote pancreatic cancer progression and gemcitabine resistance
Source: Cell Death Dis. 2025 Feb 4;16(1):68. doi: 10.1038/s41419-025-07399-1 (PMC11794584; doi:10.1038/s41419-025-07399-1)
Supplement: Supplementary file 1 — supplementary information [file 41419_2025_7399_MOESM1_ESM.pdf]

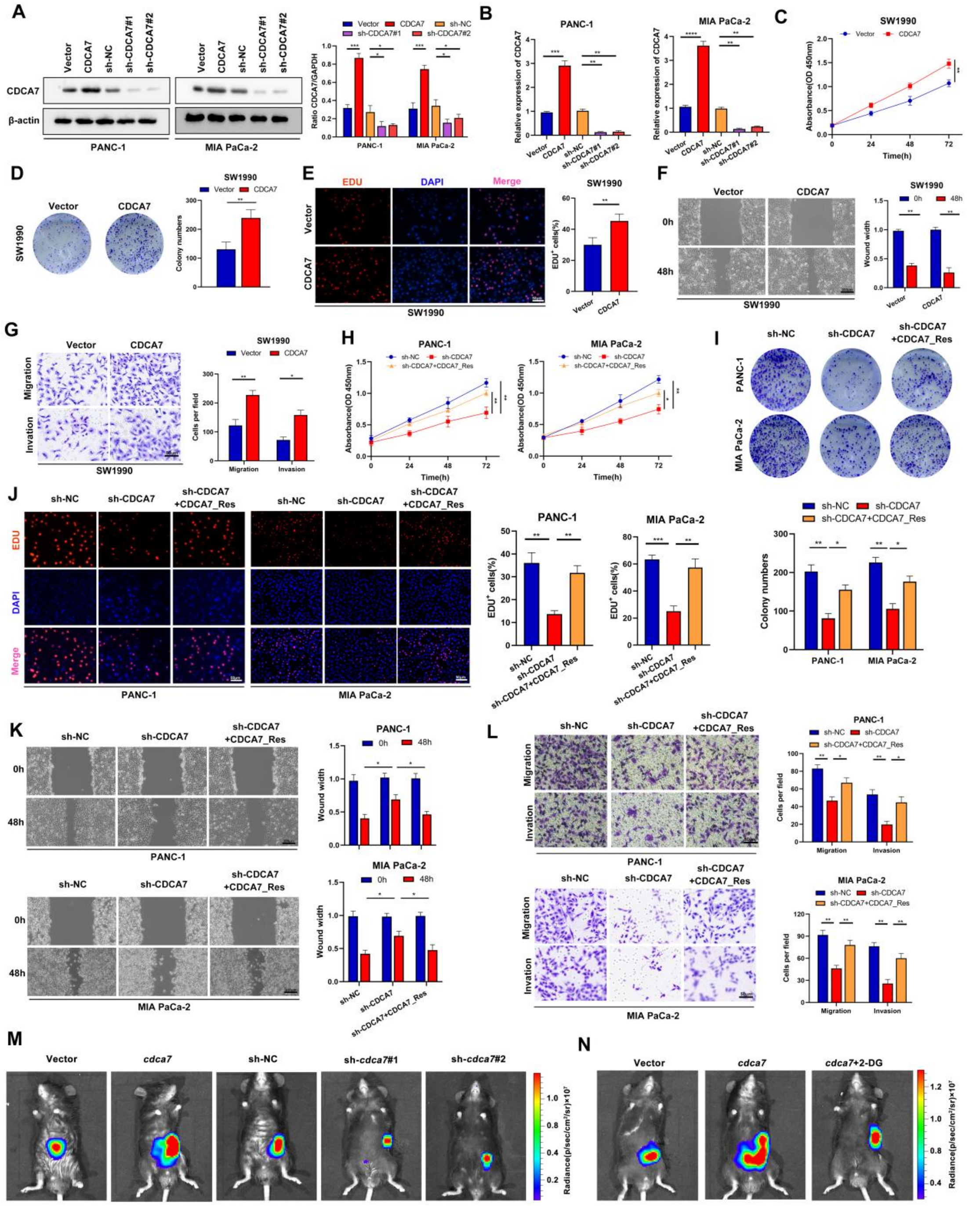

Supplementary Fig. 1. CDCA7 promotes the proliferation and migratory and invasive abilities of pancreatic cancer (PC) cells. A, B qRT-PCR and western blotting were conducted to assess CDCA7 knockdown and overexpression efficiency. (C) CCK-8, (D) plate cloning, (E) EDU assays showed increased cell proliferation. F,G Assessment of SW1990 cell migratory and invasive abilities following CDCA7 overexpression: (F) Wound healing assay, (G) Transwell assay. H–L. Reconstitution

experiment to evaluate the impact of CDCA7 modulation on cellular functions. M,N Representative bioluminescence imaging (BLI) of mice following the orthotopic injection of PANC-02 cells. Statistical significance is indicated by asterisks $*P < 0.05$, $**P < 0.01$, $***P < 0.001$.

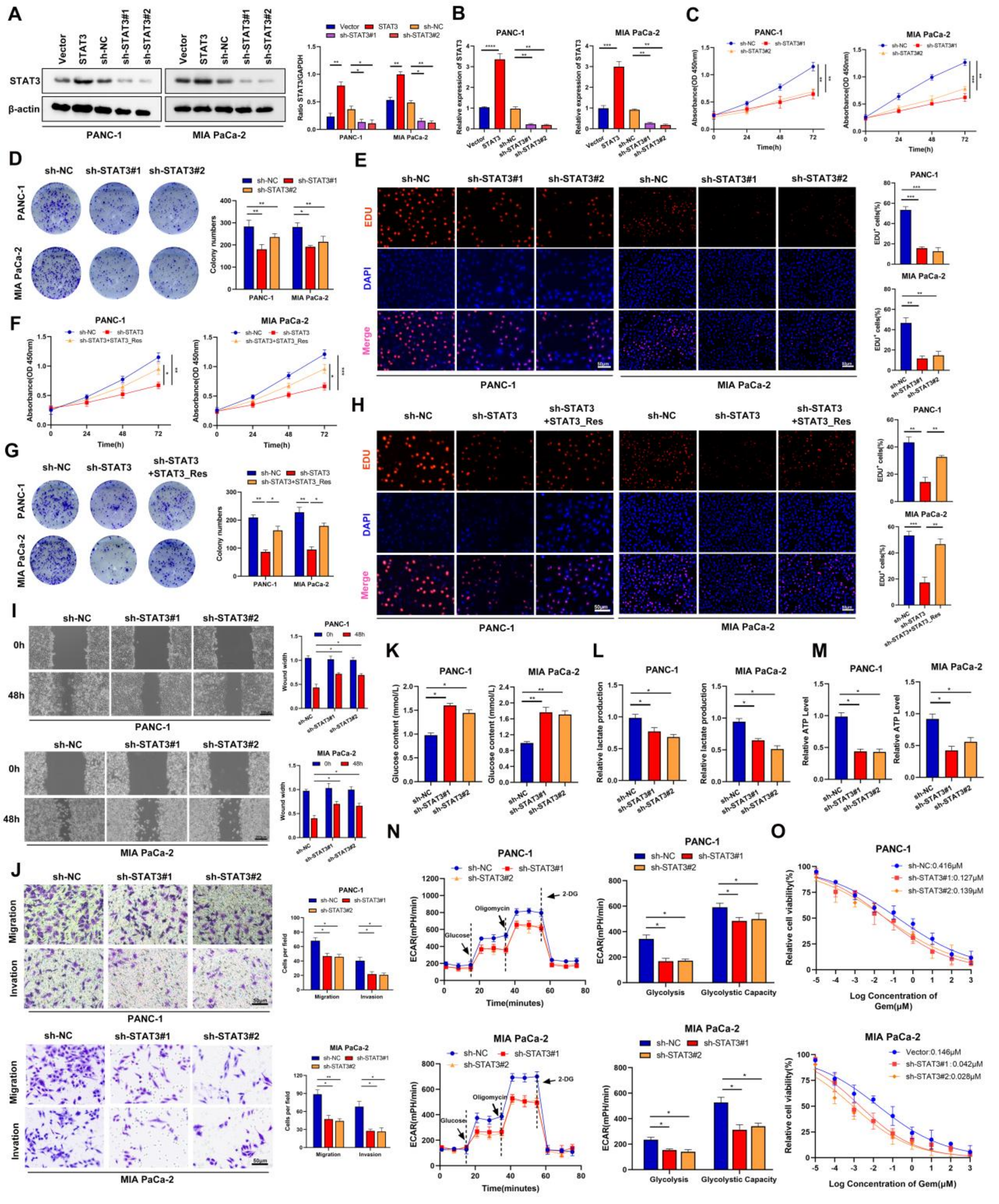

Supplementary Fig. 2. STAT3 promotes pancreatic cancer progression. A, B STAT3 knockdown or overexpression efficiency was assessed using qRT-PCR and western blotting. Cell proliferation was assessed using (C, F) CCK-8, (D, G) plate cloning, and (E, H) EDU assays. I, J The migratory and invasive abilities of STAT3-knockdown cells were assessed using scratch and Transwell assays. K–M glucose(K), Lactate (L), and ATP levels (M) in PC cells following STAT3 knockout. N Aerobic glycolysis was assessed in PC cells after STAT3 knockout, based on ECAR. O CCK8 assay was used to assess the sensitivity of PC cells to gemcitabine. $^{*}P < 0.05$,

$^{**}P < 0.01$, $^{***}P < 0.001$.

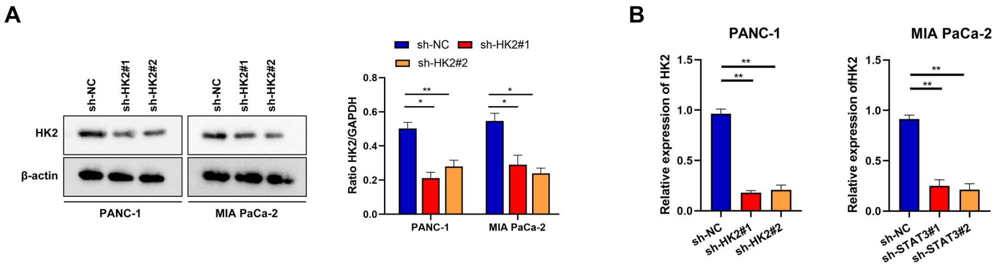

Supplementary Fig. 3. Verification of HK2 knockdown efficiency. A, B qRT-PCR and western blotting were used to examine HK2 knockdown efficiency. *$P < 0.05$, **$P < 0.01$, ***$P < 0.001$.

Supplementary Table 1 .Primers for qRT-PCR.

Primers	Forward primer 5'-3'	Reverse primer 5'-3'
GAPDH	GGAGCGAGATCCCTCCAAAAT	GGCTGTTGTCATACTTCTCATGG
CDCA7	GAGAACGTCTGCAGCAATTC	ATCCCTGACCTCTTCACCATA
STAT3	CTTTGAGACCGAGGTGTATCACC	GGTCAGCATGTTGTACCACAGG
HK2	GGTGCTGTGGCGAATCAAAG	CCTTATGGAGACGCTTGGCA
HK2-CHIP	CAGGGAGTTTGAAACTGTA	TAATCACATCCTCCATCTG

Supplementary Table 2.Catalog of Antibodies

Antibody	Vendor	Catalog number
GAPDH	Proteintech	60004-1-Ig
β-actin	Proteintech	66009-1-Ig
CDCA7	Proteintech	15249-1-AP
STAT3	Cell Signaling Technology	9139
P-STAT3	Cell Signaling Technology	9134
HK2	Proteintech	22029-1-AP
P84	Abcam	Ab487
Ki-67	Proteintech	27309-1-AP
PCNA	Proteintech	10205-2-AP

Supplementary Table 3. Sequences of shRNAs

Gene	Gene shRNA targeting sequences
Human sh-CDCA7#1	CCTCTGATGACAGTTGTGACA
Human sh-CDCA7#2	GCATATAATTCGCCCAGTGGA
mouse sh-*cdca7*#1	GCTCTCAAAGTAAAGAACTTA
mouse sh-*cdca7*#2	GAACAAAGCAATGCTTGCAAA
Human sh-STAT3#1	GCAAAGAATCACATGCCACTT
Human sh-STAT3#2	GCACAATCTACGAAGAATCAA
Human sh-HK2#1	CCAAAGACATCTCAGACATTG
Human sh-HK2#1	CCAGAAGACATTAGAGCATCT

Supplementary Table 4

Protein FDR Confidence	Gene_ID	Gene_symbol	Description	AAs	MW (kDa)	Abundance	Score
High	6774	STAT3	signal transducer and ac	770	88	1331308.25	374.2
High	6150	MRPL23	mitochondrial ribosomal	153	17.8	1374362.625	357.47
High	10963	STIP1	stress induced phosphopr	543	62.6	1365981.625	355.46
High	307	ANXA4	annexin A4	321	36.1	1364336.125	353.61
High	84864	RIOX2	ribosomal oxygenase 2	465	52.8	1362103.313	353.53
High	85313	PPIL4	peptidylprolyl isomerase	492	57.2	1361493.031	352.9
High	83743	GRWD1	glutamate rich WD repeat	446	49.4	1360513	348.92
High	64866	CDCP1	CUB domain containing pr	836	92.9	1355962.063	348.6
High	1984	EIF5A	eukaryotic translation i	184	20.2	1352961.5	346.97
High	79073	TMEM109	transmembrane protein 10	243	26.2	1351396.375	346.49
High	56902	PNO1	partner of NOB1 homolog	252	27.9	1350771.25	345.44
High	27335	EIF3K	eukaryotic translation i	218	25	1346454.625	344.47
High	2181	ACSL3	acyl-CoA synthetase long	720	80.4	1344455.625	343.06
High	5825	ABCD3	ATP binding cassette sub	659	75.4	1343955.625	340.4
High	8575	PRKRA	protein activator of int	313	34.4	1340347.063	339.03
High	79169	C1orf35	chromosome 1 open readin	263	29.4	1335840.188	334.8
High	6633	SNRPD2	small nuclear ribonucleo	118	13.5	1377058	333.83
High	51755	CDK12	cyclin dependent kinase	1490	164.1	1329630.25	332.37
High	55510	DDX43	DEAD-box helicase 43	648	72.8	1329151.625	328.62
High	30827	CXXC1	CXXC finger protein 1	660	76.2	1328829.063	319.7
High	51021	MRPS16	mitochondrial ribosomal	137	15.3	1320131.75	311.4
High	54938	SARS2	seryl-tRNA synthetase 2,	520	58	1319165.125	302.51
High	10055	SAE1	SUMO1 activating enzyme	346	38.4	1314755.25	308.65
High	84263	HSDL2	hydroxysteroid dehydroge	418	45.4	1310494.625	297.44
High	6711	SPTBN1	spectrin beta, non-eryth	2364	274.4	1305839.875	322.52
High	471	ATIC	5-aminoimidazole-4-carbo	592	64.6	1297073.625	293.54
High	28987	NOB1	NIN1 (RPN12) binding pro	412	46.6	1291154.5	299.89
High	4093	SMAD9	SMAD family member 9	467	52.5	1289201.625	318.15
High	23136	EPB41L3	erythrocyte membrane pro	1087	120.6	1286821.5	314.6
High	84061	MAGT1	magnesium transporter 1	367	41.5	1279499	312.25
High	29127	RACGAP1	Rac GTPase activating pr	632	71	1279010.094	296.36
High	6596	HLTF	helicase like transcript	1009	113.9	1278942.5	298.46
High	83879	CDCA7	cell division cycle asso	373	43	1278430.375	322.01
High	11091	WDR5	WD repeat domain 5	334	36.6	1275998.875	302.45
High	1819	DRG2	developmentally regulate	364	40.7	1274810.125	291.37
High	57727	NCOA5	nuclear receptor coactiv	579	65.5	1274295.25	289.08
High	9221	NOLC1	nucleolar and coiled-bod	699	73.6	1269747.094	285.34
High	1E+08	IGLL5	immunoglobulin lambda li	214	23	1265707.469	277.98
High	5829	PXN	paxillin	605	66.2	1260032.125	275.83
High	1890	TYMP	thymidine phosphorylase	487	50.4	1256888.25	271.44
High	29896	TRA2A	transformer 2 alpha homo	282	32.7	1252488.875	268.11
High	5784	PTPN14	protein tyrosine phospha	1187	135.2	1252327.5	265.02
High	3376	IARS1	isoleucyl-tRNA synthetas	1262	144.4	1249195.375	264.19
High	10523	CHERP	calcium homeostasis endo	916	103.6	1244876.125	262.44
High	84295	PHF6	PHD finger protein 6	365	41.3	1239879.25	257.24
High	22919	MAPRE1	microtubule associated p	268	30	1239673.625	255.32
High	121214	SDR9C7	short chain dehydrogenas	313	35.2	1238174.781	253.85
High	10095	ARPC1B	actin related protein 2/	372	40.9	1236351.375	241.56
High	11325	DDX42	DEAD-box helicase 42	938	102.9	1232138.375	240.59
High	3417	IDH1	isocitrate dehydrogenase	414	46.6	1226908.875	237.19
High	9169	SCAF11	SR-related CTD associate	1463	164.6	1220433.813	234.41
High	2801	GOLGA2	golgin A2	1002	113	1218961.875	234.01
High	55750	AGK	acylglycerol kinase	422	47.1	1218208.875	233.42
High	10713	USP39	ubiquitin specific pepti	565	65.3	1213204.25	232.5
High	5684	PSMA3	proteasome 20S subunit a	255	28.4	1212926.125	232.47
High	3964	LGALS8	galectin 8	359	40.4	1212109.125	231.57
High	9510	ADAMTS1	ADAM metallopeptidase wi	967	105.3	1208675.25	228.54
High	11128	POLR3A	RNA polymerase III subun	1390	155.5	1208226.75	228.48
High	5049	PAFAH1B2	platelet activating fact	229	25.6	1200831.25	227.56
High	1474	CST6	cystatin E/M	149	16.5	1200298.625	224.89
High	9320	TRIP12	thyroid hormone receptor	1992	220.3	1199199.844	215.21
High	970	CD70	CD70 molecule	193	21.1	1196294.75	213.73
High	4014	LORICRIN	loricrin cornified envel	312	25.7	1194535.75	213.48
High	143684	FAM76B	family with sequence sim	339	38.7	1194469.125	213.26
High	50804	MYEF2	myelin expression factor	600	64.1	1189772.75	212.2
High	10072	DPP3	dipeptidyl peptidase 3	737	82.5	1188638.625	210.56
High	9612	NCOR2	nuclear receptor corepre	2514	273.5	1187680.125	209.32
High	8666	EIF3G	eukaryotic translation i	320	35.6	1185570.406	201.16
High	9817	KEAP1	kelch like ECH associate	624	69.6	1183401.75	199.79
High	5861	RAB1A	RAB1A, member RAS oncoge	205	22.7	1182164.469	191
High	55421	NCBP3	nuclear cap binding subu	620	70.5	1182070.75	190.58
High	473	RERE	arginine-glutamic acid d	1566	172.3	1179755.625	189.92
High	6602	SMARCD1	SWI/SNF related, matrix	515	58.2	1176503.125	189.03
High	26155	NOC2L	NOC2 like nucleolar asso	749	84.9	1172248.25	188.83
High	10038	PARP2	poly(ADP-ribose) polymer	583	66.2	1168855.719	188
High	26058	GIGYF2	GRB10 interacting GYF pr	1320	152.3	1167335.469	187.46
High	3035	HARS1	histidyl-tRNA synthetase	509	57.4	1165122.875	186.5
High	10096	ACTR3	actin related protein 3	418	47.3	1162744	184.43
High	11240	PADI2	peptidyl arginine deimin	665	75.5	1159892.75	184.06
High	11335	CBX3	chromobox 3	183	20.8	1158387.625	180.92
High	22931	RAB18	RAB18, member RAS oncoge	235	26.4	1158359.125	178.7
High	55319	TMA16	translation machinery as	203	23.8	1155920.875	177.4
High	8193	DPF1	double PHD fingers 1	414	46.8	1155296.375	175.76
High	4297	KMT2A	lysine methyltransferase	3972	431.8	1153283.25	175.74
High	2950	GSTP1	glutathione S-transferas	210	23.3	1151928	172.84
High	9093	DNAJA3	DnaJ heat shock protein	480	52.5	1149579.75	171.58
High	23774	BRD1	bromodomain containing 1	1058	119.4	1147882.5	171.27
High	6923	ELOB	elongin B	161	17.9	1146217.281	171.04
High	6144	RPL21	ribosomal protein L21	160	18.6	1144889.375	170.97
High	7916	PRRC2A	proline rich coiled-coil	2157	228.7	1142068.25	170.86
High	23760	PITPNB	phosphatidylinositol tra	271	31.5	1140477.375	166.79
High	1E+08	LOC100287036	uncharacterized LOC10028	125	13.5	1140466.25	166.56
High	83444	INO80B	INO80 complex subunit B	356	38.6	1138497.125	164.27
High	4172	MCM3	minichromosome maintenan	853	95.8	1138225.5	160.88
High	6627	SNRPA1	small nuclear ribonucleo	255	28.4	1136501.25	160.61
High	90861	JPT2	Jupiter microtubule asso	190	20.1	1131250.875	160.45
High	57003	CCDC47	coiled-coil domain conta	483	55.8	1129435	159.67
High	55027	HEATR3	HEAT repeat containing 3	680	74.5	1128762.938	159.55
High	8721	EDF1	endothelial differentiat	148	16.4	1128734.125	158.73
High	427	ASAH1	N-acylsphingosine amidoh	411	46.5	1127797.063	158.38

High	1727	CYB5R3	cytochrome b5 reductase	334	38.2	1126986.625	157.76
High	64151	NCAPG	non-SMC condensin I comp	1015	114.3	1118165.25	156.33
High	64960	MRPS15	mitochondrial ribosomal	257	29.8	1118002.75	154.86
High	9184	BUB3	BUB3 mitotic checkpoint	328	37.1	1107396.125	154.06
High	338657	CENATAC	centrosomal AT-AC splici	332	38	1102798.969	153.18
High	25909	AHCTF1	AT-hook containing trans	2275	253.3	1099317.5	149.58
High	7158	TP53BP1	tumor protein p53 bindir	1977	214	1098969.813	149.13
High	55967	NDUFA12	NADH:ubiquinone oxidored	145	17.1	1096572.375	148.17
High	10632	ATP5MG	ATP synthase membrane su	103	11.4	1094639.5	146.63
High	57665	RDH14	retinol dehydrogenase 14	336	36.8	1088565.328	142.65
High	10985	GCN1	GCN1 activator of EIF2AK	2671	292.5	1081920.75	140.98
High	138046	RALYL	RALY RNA binding proteir	304	33.9	1080136	140.27
High	201626	PDE12	phosphodiesterase 12	609	67.3	1076014.5	140.02
High	10097	ACTR2	actin related protein 2	394	44.7	1072197.125	137.41
High	55631	LRRC40	leucine rich repeat cont	602	68.2	1071468.125	136.95
High	64778	FNDC3B	fibronectin type III dom	1204	132.8	1068267.125	135.3
High	10939	AFG3L2	AFG3 like matrix AAA pep	797	88.5	1068130.5	134.78
High	1173	AP2M1	adaptor related protein	435	49.6	1066520.125	134.27
High	2260	FGFR1	fibroblast growth factor	853	95.3	1065880.25	133.81
High	1781	DYNC1I2	dynein cytoplasmic 1 int	638	71.4	1065687.875	132.4
High	6396	SEC13	SEC13 homolog, nuclear p	322	35.5	1065581.406	131.34
High	7706	TRIM25	tripartite motif contair	630	70.9	1064562.625	131.14
High	7453	WARS1	tryptophanyl-tRNA synthe	471	53.1	1063692.5	131.06
High	30001	ERO1A	endoplasmic reticulum ox	468	54.4	1062674.094	130.55
High	3098	HK1	hexokinase 1	921	102.7	1062558.031	128.74
High	667	DST	dystonin	5675	649.1	1061084.281	128.44
High	5691	PSMB3	proteasome 20S subunit b	205	22.9	1060512.688	128.44
High	57602	USP36	ubiquitin specific pepti	1123	122.8	1060295.25	127.93
High	10960	LMAN2	lectin, mannose binding	356	40.2	1056085	126.56
High	29105	CFAP20	cilia and flagella assoc	193	22.8	1055736.219	124.6
High	23234	DNAJC9	DnaJ heat shock protein	260	29.9	1055645.5	124.42
High	10328	EMC8	ER membrane protein comp	210	23.8	1055455.125	123.4
High	83659	TEKT1	tektin 1	418	48.3	1049304.875	123.27
High	22894	DIS3	DIS3 homolog, exosome er	958	108.9	1047696	122.81
High	2109	ETFB	electron transfer flavop	346	37.4	1047158.875	120.9
High	65008	MRPL1	mitochondrial ribosomal	325	36.9	1046742.25	120.82
High	1278	COL1A2	collagen type I alpha 2	1366	129.2	1044892.188	120.52
High	26017	FAM32A	family with sequence sim	112	13.2	1041594.438	119.15
High	1445	CSK	C-terminal Src kinase	450	50.7	1040184.188	118.03
High	27445	PCLO	piccolo presynaptic cytc	5142	560.4	1037069.438	117.29
High	1763	DNA2	DNA replication helicase	1060	120.3	1035491.563	116.81
High	58503	OPRPN	opiorphin prepropeptide	248	27.2	1033942.125	116
High	2584	GALK1	galactokinase 1	392	42.2	1033214.813	115.62
High	3839	KPNA3	karyopherin subunit alph	521	57.8	1028900.188	115.49
High	26092	TOR1AIP1	torsin 1A interacting pr	584	66.3	1023460.688	115.3
High	55544	RBM38	RNA binding motif protei	239	25.5	1023229.563	114.57
High	29097	CNIH4	cornichon family AMPA re	139	16.1	1022163.125	114.55
High	445	ASS1	argininosuccinate syntha	412	46.5	1021333.156	111.79
High	16	AARS1	alanyl-tRNA synthetase 1	968	106.7	1021192.313	110.86
High	1537	CYC1	cytochrome c1	325	35.4	1014449.438	110.56
High	5091	PC	pyruvate carboxylase	1178	129.6	1013840.563	110.49
High	37	ACADVL	acyl-CoA dehydrogenase v	678	72.9	1013791.375	110.07
High	10054	UBA2	ubiquitin like modifier	640	71.2	1011204.938	110.02
High	1627	DBN1	drebrin 1	649	71.4	1007253.438	108.99
High	10241	CALCOCO2	calcium binding and coil	470	55.2	1003886.75	108.65
High	388610	TRNP1	TMF1 regulated nuclear p	227	23.4	1002669.5	107.63
High	55127	HEATR1	HEAT repeat containing 1	2144	242.2	1001179.813	107.25
High	25949	SYF2	SYF2 pre-mRNA splicing f	243	28.7	1001140.063	107.23
High	5859	QARS1	glutaminyl-tRNA syntheta	775	87.7	995744.0625	106.51
High	5604	MAP2K1	mitogen-activated protei	393	43.4	995052.4688	106.01
High	4646	MYO6	myosin VI	1285	148.6	993106.2813	105.96
High	10849	POLR1G	RNA polymerase I subunit	510	55	992472.75	103.28
High	56922	MCCC1	methylcrotonyl-CoA carbo	725	80.4	990649.0625	102.21
High	79902	NUP85	nucleoporin 85	656	75	990313.9375	102.06
High	6301	SARS1	seryl-tRNA synthetase 1	514	58.7	987336.5625	100.65
High	84659	RNASE7	ribonuclease A family me	156	17.4	987125.125	100.57
High	58517	RBM25	RNA binding motif protei	843	100.1	985343.5625	100.54
High	6723	SRM	spermidine synthase	302	33.8	984820.8125	98.893
High	643834	PGA3	pepsinogen A3	388	42	984099.4375	97.752
High	6603	SMARCD2	SWI/SNF related, matrix	531	58.9	978786.375	97.231
High	55276	PGM2	phosphoglucomutase 2	612	68.2	978140.8125	95.702
High	1983	EIF5	eukaryotic translation i	431	49.2	977735.1875	94.235
High	998	CDC42	cell division cycle 42	191	21.2	974770.3125	93.835
High	5717	PSMD11	proteasome 26S subunit,	422	47.4	969155.25	93.08
High	9879	DDX46	DEAD-box helicase 46	1031	117.3	963721.5625	92.374
High	79577	CDC73	cell division cycle 73	531	60.5	960425.8125	92.149
High	51575	ESF1	ESF1 nucleolar pre-rRNA	851	98.7	957204	92.049
High	3934	LCN2	lipocalin 2	198	22.6	956536.5625	90.262
High	6237	RRAS	RAS related	218	23.5	951084.625	89.519
High	5284	PIGR	polymeric immunoglobulir	764	83.2	949753.5625	87.091
High	114049	BUD23	BUD23 rRNA methyltransfe	281	31.9	948260.375	84.282
High	5496	PPM1G	protein phosphatase, Mg2	546	59.2	946051.4375	82.406
High	51635	DHRS7	dehydrogenase/reductase	339	38.3	944019.3125	81.7
High	5701	PSMC2	proteasome 26S subunit,	433	48.6	943761.1875	80.435
High	23478	SEC11A	SEC11 homolog A, signal	185	21.4	943758.8125	77.628
High	6709	SPTAN1	spectrin alpha, non-eryt	2477	284.9	940528.5625	76.287
High	54865	GPATCH4	G-patch domain containir	375	42.6	936986.375	67.292
High	1982	EIF4G2	eukaryotic translation i	907	102.3	936472.5938	57.603
High	3735	KARS1	lysyl-tRNA synthetase 1	625	71.5	932611.9375	45.298
High	8453	CUL2	cullin 2	764	89.4	928006	43.718
High	4258	MGST2	microsomal glutathione S	147	16.6	927835.0625	26.122
High	1106	CHD2	chromodomain helicase DN	1828	211.2	927128.625	23.031
High	23597	ACOT9	acyl-CoA thioesterase 9	448	50.8	925493.4063	20.857
High	9588	PRDX6	peroxiredoxin 6	224	25	922290.5625	20.849
High	2739	GLO1	glyoxalase I	184	20.8	922109.6875	14.093
High	10811	NOXA1	NADPH oxidase activator	483	51.6	919297.25	9.7042
High	6836	SURF4	surfeit 4	269	30.4	917535.5	3.6417
