# Supplementary figures and images for: CDCA7 enhances STAT3 transcriptional activity to regulate aerobic glycolysis and promote pancreatic cancer progression and gemcitabine resistance
Source: Cell Death Dis. 2025 Feb 4;16(1):68. doi: 10.1038/s41419-025-07399-1 (PMC11794584; doi:10.1038/s41419-025-07399-1)

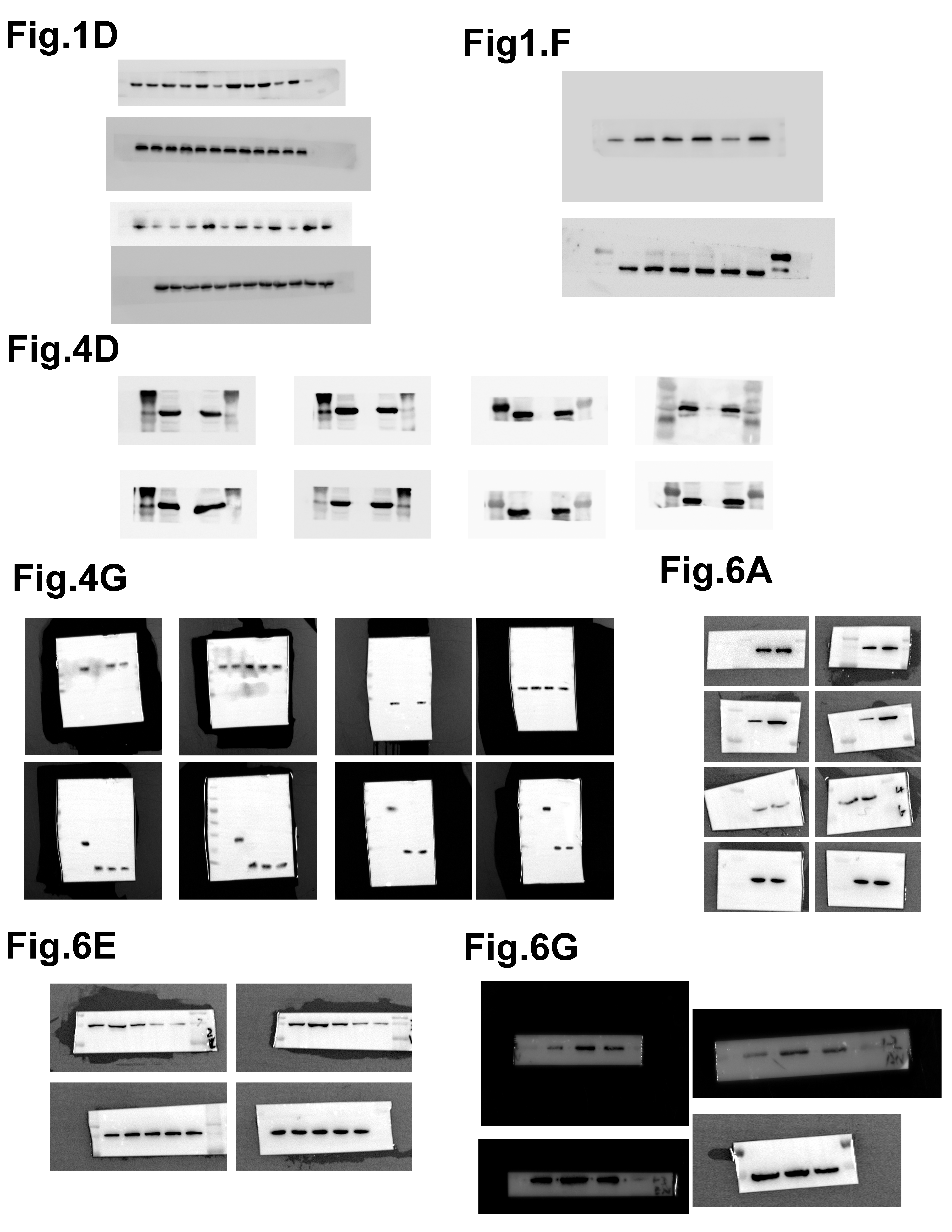

Supplement: Supplementary file 2 — Original Data1 [file 41419_2025_7399_MOESM2_ESM.tif]

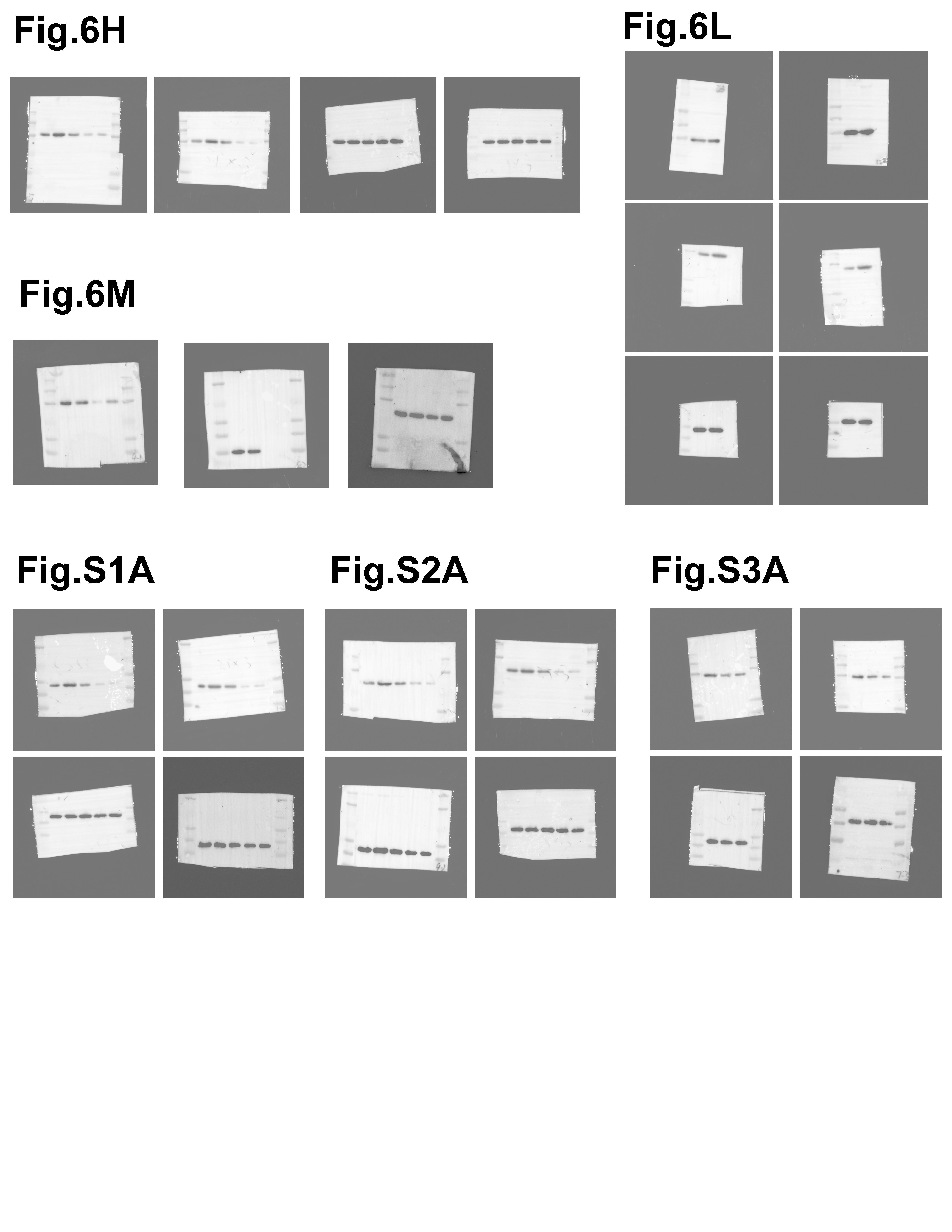

Supplement: Supplementary file 3 — Original Data2 [file 41419_2025_7399_MOESM3_ESM.tif]
